# *Legionella longbeachae* and Endocarditis

**DOI:** 10.3201/eid1801.110579

**Published:** 2012-01

**Authors:** Nicola Leggieri, Frédérique Gouriet, Frank Thuny, Gilbert Habib, Didier Raoult, Jean-Paul Casalta

**Affiliations:** Centre Hospitalier-Universitaire de La Timone, Marseille, France

**Keywords:** endocarditis, Legionella longbeachae, potting mixes, respiratory infections, bacteria

## Abstract

We report a case of infectious endocarditis attributable to *Legionella longbeachae*. *L. longbeachae* is usually associated with lung infections. It is commonly found in composted waste wood products. *L. longbeachae* should be regarded as an agent of infectious endocarditis, notably in the context of gardening involving handling of potting soils.

*Legionella longbeachae* is a facultative intracellular gram-negative bacillus commonly found in composted waste wood products used in potting mixes. It is usually associated with lung infections. We report a case of infectious endocarditis attributable to *L. longbeachae* 6 months after the patient had an aortic valve bioprothesis replacement.

## The Patient

In July 2008, a 73-year-old man was admitted to La Timone Hospital, Marseille, France; it was suspected that an aortic bioprosthetic valve had become displaced. The patient had received an aortic bioprosthetic valve replacement in January 2008 for aortic insufficiency. After he returned home in March, he carried out gardening activities and used potting mixes to plant flowers. In April 2008, he was admitted to the emergency department of a general hospital for fever (38.9°C). At admission, his leukocyte count was 12 × 10^9^ cells/L. He was empirically treated with amoxicillin/clavulanic acid plus ciprofloxacin. Results of clinical examination and all investigations (chest radiograph, transesophageal echocardiograph, 3 blood cultures, urine analysis, and *Legionella* urinary antigen test) did not identify an infectious agent, and antimicrobial drug treatment was stopped after 24 hours.

In May 2008, the patient had a new episode of fever (39°C), with a weight loss of 20 kg within 3 months. He was admitted to the cardiology department of La Timone Hospital. An endocarditis diagnostic kit was used. The endocarditis kit included, besides blood cultures, tubes to collect a serum sample, which was used to detect rheumatoid factor and estimate specific antibodies against *Coxiella burnetii, Bartonella* spp., *Brucella* spp., *Chlamydia* spp., *Mycoplasma pneumoniae*, *Legionella pneumophila, L. anisa*, and *Aspergillus* spp (*1*). Results of all tests (including transesophageal echocardiograph) were negative. The modified Durack score (*2*) showed that only 2 minor criteria were met (and no major criteria), and the Richet score was 3 (fever = 1, male = 1, previous valvular pathology = 1), with a positive predictive value of 0.28 and a negative predictive value of 0.82 (*3*). He was discharged without receiving any antimicrobial drugs.

In July 2008, the patient was hospitalized at La Timone Hospital for heart failure. A transesophageal echocardiograph showed an aortic bioprothesis displacement with a false aneurysm and 9 mm of vegetation (Figure). At admission, leukocyte count was 9.4 × 10^9^ cells/L, hemoglobin level was 9.8 gm/dL, erythrocyte sedimentation rate was 53 mm, and C-reactive protein level was 126 mg/L. A new endocarditis diagnostic kit was used. The blood cultures remained sterile. Results of serologic tests for *L. pneumophila* and the urinary antigen test for *Legionella* spp. were negative. Only the serologic test for *L. anisa* was positive (titer 256). The diagnosis of blood culture–negative endocarditis was established, and intravenous antibiotherapy was begun with vancomycin (30 mg/kg/d) plus gentamicin (3 mg/kg/d).

**Figure:**
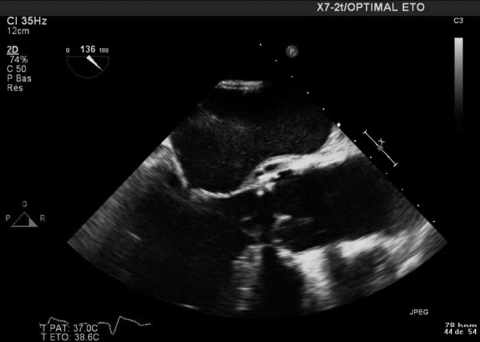
Transesophageal echocardiograph of patient with Legionnaires’ disease, Marseille, France, July 2008.

Cardiac surgery was performed after 30 days because of heart failure. The aortic bioprothesis was replaced. We conducted bacterial 16S rDNA amplification and sequencing on the valvular tissue as reported (*4*) and found a sequence 100% similar to that of *L. longbeachae* (GenBank accession no. AY444741). The valvular culture grew gram-negative bacilli that were catalase positive and oxidase negative after 10 days on buffered charcoal yeast extract medium (AES Chemunes, Bruz, France). The strain was identified as *L. longbeachae* by 16S rDNA (*5*) and *mip* gene (*6*) amplification and sequencing (GenBank accession no. AJ810226).

Histologic examination of the removed valve was performed, and findings were compatible with the diagnosis of infective endocarditis. Results of immunohistochemical analysis performed on the valvular tissue were positive for *L. longbeachae*. Results of immunofluorescence and Western blot of the patient’s blood were positive for the same strain.

The patient recently used only Australian potting mixes, which may contain *L. longbeachae*, although we were not able to cultivate *L. longbeachae* or other *Legionella* spp. from the potting mix. Vancomycin administration was stopped, and the patient’s treatment was changed to erythromycin (3 g/d intravenously for 6 weeks) and ciprofloxacin (400 mg/d intravenously for 15 days and 1.5 g/d by mouth for 1 month). The patient recovered. At a 3-month follow-up visit, he had gained 6 kg, and no inflammatory syndrome was found. At 6 months, he had gained 13 kg and felt well.

## Conclusions

*L. longbeachae* was first described as a new species in 1981 after it was isolated from a patient with pneumonia in Long Beach, California, USA (*7**–**9*). A second serogroup was described in 1981 (*10*). This species is common in composted wood products used in potting mixes in Australia and in Japan (*11*,*12*). Although the composition of potting soil used in Europe is different, Legionnaires’ disease has also been described in Scotland in connection with the use of potting soil (*13*). A case of pneumonia caused by *L. longbeachae* associated with gardening was described in 2006 in the Netherlands. *L. longbeachae* was cultured from the patient’s sputum and from the commercial potting soil he had used (*14*). The source of contamination for the patient described could be the Australian potting mixes he had used for 1 month before the first symptoms, although a culture of potting soil was negative. The patient did not experience cutaneous inoculation or a wound during gardening, although minor trauma is a possibility.

Noninvasive diagnosis for this patient was not successful: the results of serologic immunofluorescence testing for *L. pneumophila* and the urinary antigen test for *Legionella* spp. were negative. Only the serologic testing was positive for *L. anisa* (titer 256). Cross-reactivity between *L. anisa* and *L. longbeachae* should be considered.

In a previous report, a patient with aortic valve endocarditis showed an increase in titers of antibodies against *L. bozemanii*, *L. longbeachae*, and *L. jordanis*, but the infection was not confirmed by the isolation of bacteria (*15*). The patient was a 38-year-old woman with type 1 diabetes mellitus and bronchial asthma but no medical history of heart disease. She was hospitalized 2 months before acquiring infectious endocarditis for pneumonia and fever caused by *M. pneumoniae*. Transesophageal echocardiograph showed a thickened bicuspid aortic valve with 5–7-mm vegetation. The serologic test results were positive only for *L. bozemanii*, *L. longbeachae*, and *L. jordanis*. She was treated with erythromycin (1 g 4×/d intravenously, then orally, for 6 weeks, then 1 g 3×/d orally for 2 weeks). For the patient reported here, the diagnosis of infectious endocarditis was definitive because results of PCR, serologic testing, and valvular culture were positive for *L. longbeachae*.

*L. longbeachae* should be regarded as an agent of infectious endocarditis, notably in context of gardening involving handling potting soils. Definitive identification was possible only by using molecular biology–based methods on the removed valve and on culture. As mentioned by other investigators, *L. pneumophila* urinary antigen test should not be used to rule out *L. longbeachae* infection (*13*).
